# Open Online Courses in Public Health: experience from Peoples-uni

**DOI:** 10.12688/f1000research.10728.2

**Published:** 2017-04-28

**Authors:** Richard F. Heller, Robert Zurynski, Alan Barrett, Omo Oaiya, Rajan Madhok

**Affiliations:** 1 People’s Open Access Education Initiative (Peoples-uni), Manchester, UK; 2 Consulting Services Pty Ltd, Concord West, Australia; 3 West and Central African Research and Education Network, Accra, Ghana

**Keywords:** online learning, developing country, Public Health, open online courses

## Abstract

Open Online Courses (OOCs) are offered by Peoples-uni at http://ooc.peoples-uni.org to complement the courses run on a separate site for academic credit at http://courses.peoples-uni.org. They provide a wide range of online learning resources beyond those usually found in credit bearing Public Health courses. They are self-paced, and students can enrol themselves at any time and utilise Open Educational Resources free of copyright restrictions.  In the two years that courses have been running, 1174 students from 100 countries have registered and among the 1597 enrolments in 14 courses, 15% gained a certificate of completion. Easily accessible and appealing to a wide geographical and professional audience, OOCs have the potential to play a part in establishing global Public Health capacity building programmes.

## Introduction

Peoples-uni was developed with the mission “To contribute to improvements in the health of populations in low- to middle-income countries by building Public Health capacity via e-learning at very low cost” ( http://www.peoples-uni.org/content/overall-objectives) ^1, 2^. From 2008, formal courses have been run online, and it has been possible for students to gain academic credit towards a Master of Public Health award. A small fee is charged and an international group of volunteer tutors from 51 countries facilitate online discussions and set and mark assignments – to date 1256 people, from 80 countries have enrolled, 464 have passed at least one module and 111 have graduated with a Master of Public Health. With the aim of extending these offerings, reaching a wider audience and contributing further to global health and health system strengthening, an n additional site was established for free Open Online Courses (OOCs) in 2014 ( http://ooc.peoples-uni.org). This educational innovation is also designed to contribute to leadership development through lifelong learning among health professionals. While there are similarities with Massive Open Online Courses (MOOCs), there are a number of differences, including resources to be read rather than video recorded lectures. This report summarises the experience so far with Open Online Courses (OOCs).

## Methods

A number of courses were developed and placed on the Moodle open source educational platform at http://ooc.peoples-uni.org. Development of the site was led by RFH (first author of this paper), in consultation with other volunteers from Peoples-uni, and in response to requests from external organisations and individuals. Choice of the courses was to some extent serendipitous, and depended on an assessment of gaps in conventional courses and opportunities to provide courses for, and receive input from, others. Most of courses were developed by Peoples-uni volunteer academics and IT support staff, with input and review from various experts to ensure relevance of the course material. One of the courses reported here was developed using e-learning course materials from the University of Nottingham (Basic Epidemiology), two others were developed for the UK Global Health Exchange ( http://www.globalhealthexchange.co.uk), to provide basic Public Health knowledge to health professionals planning to volunteer overseas, another to provide open access for educational materials developed by an international collaboration in exercise and health, and another by an international collaboration in medical ethics.

Access is by self-enrolment with nomination of a username and password for future use. All resources are Open Educational Resources, free of copyright restrictions. A common format is used with learning objectives, links to or copies of key parts of online resources, and metadata to direct students through the resources. Rather than online discussions facilitated by tutors (as in the Peoples-uni academic stream and many MOOCs), questions on the content and implications of the resources are posed for students to reflect upon, and forums are enabled for students to post these reflections for other students to see. Quizzes were developed to test the knowledge gained, and a certificate of completion is automatically generated if various criteria are met such as accessing resources, completing the quizzes, posting to a forum or providing feedback. There is no specified timetable and students pace themselves through the course. There is no charge for enrolment or for the certificate. Courses are published under a Creative Commons Attribution 4.0 International License.

Information about the courses was offered to students and graduates of the Peoples-uni academic stream and was posted on various social media sites. Two courses were provided to participants planning to travel overseas through the Global Health Exchange, and in one case information was distributed to deans of Australian and New Zealand medical schools to encourage medical students to learn about the Public Health implications of climate change.

We report here the first two years of experience with the Peoples-uni Open Online Courses, including information from the questions asked on registration about student demographics and how they planned to access the courses. Formal feedback was not a requirement generally, although some courses provided the option for feedback, and we report some of these comments. No ethical approval was required for publication of de-identified student demographics.

### Statistical analysis

Data on student demographics at registration were obtained by SQL enquiries using the configurable report facility in Moodle. Data on whether the student had obtained a certificate were obtained by an SQL query against the Moodle database, supplemented by course data obtained from the course databases in Moodle. Descriptive analysis of frequency counts was performed using the R statistical package.

## Results

The data reported here relate to those who enrolled as students on 14 self-paced courses up to December 2016, with variable start dates from June 2014. 1174 students registered, from 100 countries - some of these students enrolled in more than one of the 14 courses available and we report on 1597 course enrolments by these students. Table 1 shows the number of students per course, the number who gained a certificate of completion, and the criteria for a certificate. Courses have been added over the years, so the time period over which students can enrol varies. The criteria for the award of a certificate can be seen to vary, and although the overall percentage of students that were awarded certificates was 15%, there was some small variation between courses. Seven students each enrolled in 9 courses or more – they were responsible for 49 (20%) of the 243 certificates gained.

**Table 1.** Numbers of students enrolled in each course, and the number of students who gained a certificate. The criteria for a certificate are also shown.

Course title	N students	N (%) gained a certificate	Criteria for a certificate
Public Health – for the GHE *	292	53(18%)	Take 4 quizzes
Public Health - the basics **	52	8 (15%)	Access 7 sets of resources, take quiz
Disease in developing countries **	18	6 (33%)	Access 6 sets of resources, take quiz
Prevention – for the GHE *	80	13 (16%)	Access 3 sets of resources, post 3 reflections
Basic Epidemiology from the University of Nottingham	404	59 (15%)	Access 8 sets of resources, pass quiz
Climate change and Public Health ***	212	23 (11%)	Access 7 sets of resources, take 2 quizzes
Refugee health	144	24 (17%)	Access 6 sets of resources
Human rights and Public Health	53	10 (19%)	Access 6 sets of resources, send feedback
Exercise and health	54	3 (6%)	Post 4 reflections, pass 1 quiz
Medical ethics	62	9 (15%)	Take 5 quizzes, send feedback
Medical professionalism ****	85	16 (19%)	Access 5 resources, take 1 quiz
Clinico-epidemiology conference ****	72	10 (14%)	Access 6 sets of resources, take 1 quiz
Global Health Informatics	47	9 (19%)	Access 6 sets of resources, take quiz
Patient Safety	22	0	Access 11 sets of resources and take 11 quizzes
Total	1597	243 (15%)	

* : Offered to participants in the Global Health Exchange programme ** : Later subdivisions of the Public Health for the GHE course *** : Information about launch of this module sent to deans of Australian and NZ medical schools **** : Previously offered as timetabled courses with online facilitated discussions

As part of the enrolment process, a number of questions were asked of the students. The responses are shown in Table 2 and Table 3. Table 2 shows that the largest single group of students came from Africa. Students were evenly distributed between males and females, mostly born between 1970 and 1989; 58% were health professionals and 25% were students. Table 3 shows that the majority of students enrolled as a result of a recommendation and that this would be their first experience of online learning (63 and 68% of those responding, respectively). Only 20% found the site by an internet search, the majority would be able to spend only up to 2 hours a week on the course, although 21% of those responding claimed to be able to spend 4 hours or more per week. The majority of those responding, 83%, planned to access the courses by computer rather than phone or tablet.

**Table 2.** Demographics of 1174 students registered in Peoples-uni Open Online Courses.

Demographics		Numbers (% of those with information)
Gender	Male	551 (49%)
	Female	575 (51%)
	Not specified	48
Date of birth	1940–1959	94 (8%)
	1960–1969	161 (14%)
	1970–1979	284 (25%)
	1980–1989	413 (36%)
	1990 or later	193 (17%)
	Not specified	29
Location	Africa	294 (27%)
	Australia/New Zealand	255 (24%)
	UK	197 (18%)
	Indian subcontinent	131 (12%)
	North America/Europe	110 (10%)
	Asia/Middle East// Latin America	93 (9%)
	Other/Not specified	94
Occupation	Medical practitioner	314 (28%)
	Nurse/Other health professional	338 (30%)
	Other	186 (17%)
	Student	282 (25%)
	Not specified	54

**Table 3.** Information from 1174 students registered on Peoples-uni Open Online Courses.

Question	Response	Number (% of those with information)
How did you hear about the course	Recommended by someone else *	527 (63%)
	Searching the web	164 (20%)
	Other	146 (17%)
	Not specified	337
Previous study	Previous MOOC or online course	142 (21%)
	Previous Peoples-uni student	77 (11%)
	First time on this type of online course	473 (68%)
	Not specified	482
Time you can spend per week	Less than 1 hour	115 (14%)
	1–2 hours	349 (41%)
	3–4 hours	202 (24%)
	4 hours or more	181 (21%)
	Not specified	327
Will access courses	Mostly via phone	79 (9%)
	Mostly via tablet	68 (8%)
	Mostly via computer	724 (83%)
	Not specified	303

* : Includes access recommended by the Global Health Exchange

Some comments from feedback forms are shown in Box 1. The responses were generally positive, although suggestions for improvement were made. Four additional courses were developed in partnership with other organisations, and were offered with a timetable of expert tutors facilitating online discussions. 127 students enrolled in these, of which 18 (14%) gained a certificate. Two of the courses were later adapted as self-paced versions, and appear in the course list in Table 1 in their later iterations.

Box 1. **Comments taken from feedback forms included in some of the courses.**

“The idea was great. Its an easy to learn method faster and quite informative.”

“very interesting and fruitful courses”

“Thank you for the course. It is a broad overview of may different areas in medical ethics.”

“Really enjoyed the course - very interesting”

“Good outline and overview of selected topics”

“overall is a good course”

“It is a very good course, and I am very happy with the results.”

“The course is well structured.”

“I have taken previous courses in these modules offered online and this one seems a bit too hands-off for a course. It reads more like a manual that certainly attracts interested people but does not provide overly a learning experience. I presume that mini quizzes, crossroads-exercises that block advancement unless completed and the like would create a teaching scenario better.”

“this course has been very very rewarding. it has enlightening my knowledge knowing that the Public Health is essential in line with the rights of everyone involved. I believed that more emphasis be made on low-income countries like mine (Liberia).”

“Great course - really enjoyed it”

“i am working in Central African Republic and I am an immunization specialist. I am working with …... and I think that after this course, it mandotory for me to make sure that evrychldren in the refugees camp get his polio vaccines correctly.”

“what I like most about this course was the simple break down in the course delivery, and the in which all lessons were well structured, also a clear explanation of every terminology,”

“The course was good, very basic and a good introduction.”

“short and informative for a basic introduction to the difference aspects of public health”

“Its very educative”

## Discussion

Our experience demonstrates that a volunteer-led organisation can develop and offer OOCs which are accessible by a global audience. A wide range of topics have been covered, beyond those usually found in award courses in Public Health, and more courses have since been posted on the site further to those included in this report, whilst others are under development.

Students were equally spread between genders, mostly aged around 25–40, and included a high proportion from developing countries. Certificates were gained by 15% of participants, and there were no obvious differences in course characteristics that explained the small variation in proportion of participants gaining these certificates between courses. The major predictor of gaining a certificate among those we examined was the number of courses taken by a student, with just 7 students gaining 20% of the certificates.

The qualitative feedback reported here is selective and may well not be representative of the general experience of the students, however the majority were positive about their experiences. We are utilising both the positive feedback and constructive suggestions to work to improve the course experience.

The format for the Peoples-uni Open Online Courses differs from that of MOOCs in a number of ways, although the basic methodology of online learning remains the same. The courses we report here contain mainly written content with hyperlinks to the resources, rather than the ‘talking head’ videos which are the staple of MOOCs (although this reliance on video lectures has been criticised ^3^). This allows us to utilise Open Educational Resources (OER) ^4^ and access excellent educational material instead of having to develop it anew. In contrast to the usual MOOCs, students can enrol at any time, there is no specified timetable and students pace themselves through the course. Forums are available, but designed for reflection rather than discussion, and a certificate of completion is available according to various criteria such as taking a quiz and downloading resources (see Table 1). Our model excludes interaction between students and tutors, but allows greater flexibility in timing and access to education.

MOOCs have been offered by many educational organisations. The majority of their students are from North America or Europe, an experience common to most ^5^. The Johns Hopkins School of Public Health has a long history of open access education, and they report experience with a number of MOOCs ^6^. The School reports a median completion rate of 11% ^6^, consistent with 12.6% reported by Jordan ^7^ and higher than the Coursera experience of 4% ^5^. To date, we have had approximately half of our students from developing countries. Our certification rate of 15% is consistent with the MOOC experience, although not many comparisons can be made in terms of course length, complexity, audiences and topics.

MOOCs have been subdivided into xMOOCs, based on traditional university courses but without teacher-student interactions, and cMOOCS where collectivists of teachers and learners work together to explore content ^8^. There are a number of other described variants, of which Self Paced Open Courses (SPOCs) are most closely related to the Peoples-uni type of course ^9^.

In pedagogical terms, those who enrol on the OOC courses are independent learners, who will be exposed to the hierarchy of thinking reflected by knowledge, comprehension and application in Bloom’s Taxonomy. While the methodology used provides students a view of Bloom’s higher levels of analysis, synthesis and evaluation, we recognise that without in depth student/teacher interaction it is unlikely that competence in these areas will be achieved, and they are not tested by the quizzes we have used to assess the educational outcomes. The OOC approach only aims to provide an environment for learning, rather than adding student/teacher interactions, thus could be considered in traditional learning theory as cognitivism, although the newer term, connectivism, might be a better reflection given the digital nature of the learning environment ^10^.

Based on our experience, it would appear that the Peoples-uni type of programme has a place on the educational spectrum. We see OOCs as being a major component of a modern framework for public health capacity building through global learning. The approach responds to current worldwide pressures in public health and workforce development to use low-cost models based on online learning, international volunteer tutors, teaching throughout career progression, and providing timely and appropriate content. We recognise the limitations of the descriptive analysis presented here as a measure of effectiveness, and hope to be able to design and perform a more rigorous evaluation of this educational approach.

We have also offered this platform to other providers and, in keeping with the social enterprise model of Peoples-uni, have developed courses for other organisations and their audiences. There are currently more than 20 courses available on our site; we welcome others who wish to utilise this platform in collaboration.

## Conclusions

Open Online Courses, offered by Peoples-uni on http://ooc.peoples-uni.org to complement the courses run on a separate site for academic credit on http://courses.peoples-uni.org, provide a wide range of online learning beyond that usually found in credit bearing Public Health courses. Accessible to a wide geographical and professional audience, and providing a certificate to those who persist in the learning process, they complement MOOCs in being available for self-paced learning at any time. They have the potential to play a part in establishing global Public Health capacity building programmes.

De-identified data collected showing numbers of students at Peoples-uni enrolled in each course, and the number of students who gained a certificate, from June 2014 to December 2016 These data were used to create Table 1. Click here for additional data file. Copyright: © 2017 Heller RF et al. 2017 Data associated with the article are available under the terms of the Creative Commons Zero "No rights reserved" data waiver (CC0 1.0 Public domain dedication).

De-identified data collected on student demographics at Peoples-uni from June 2014 to December 2016 These data were used to create Table 2 and Table 3. Click here for additional data file. Copyright: © 2017 Heller RF et al. 2017 Data associated with the article are available under the terms of the Creative Commons Zero "No rights reserved" data waiver (CC0 1.0 Public domain dedication).

## Data availability

The data referenced by this article are under copyright with the following copyright statement: Copyright: © 2017 Heller RF et al.

Data associated with the article are available under the terms of the Creative Commons Zero "No rights reserved" data waiver (CC0 1.0 Public domain dedication). 

**Dataset 1: De-identified data collected showing numbers of students at Peoples-uni enrolled in each course, and the number of students who gained a certificate, from June 2014 to December 2016.** These data were used to create Table 1.

DOI, 10.5256/f1000research.10728.d151763 ^11^

**Dataset 2: De-identified data collected on student demographics at Peoples-uni from June 2014 to December 2016.** These data were used to create Table 2 and Table 3.

DOI, 10.5256/f1000research.10728.d151764 ^12^
