# Supplementary material for: Open Online Courses in Public Health: experience from Peoples-uni
Source: F1000Res. 2017 Apr 28;6:170. Originally published 2017 Feb 21. [Version 2] doi: 10.12688/f1000research.10728.2 (PMC5399966; doi:10.12688/f1000research.10728.2)
Supplement: Peer review history

# Peer review: Referee response for version 2

**DOI:** 10.5256/f1000research.12436.r22334

**Published:** 2017-05-08

**Authors:** Michael Rowe

**Affiliations:** 1 University of the Western Cape, Department of Physiotherapy, Faculty of Community and Health Sciences, Bellville, South Africa

I believe that the authors have addressed the comments I made on the previous version of the note.

I have read this submission. I believe that I have an appropriate level of expertise to confirm that it is of an acceptable scientific standard.

# Peer review: Referee response for version 2

**DOI:** 10.5256/f1000research.12436.r22465

**Published:** 2017-05-03

**Authors:** Jane-frances Obiageli Agbu

**Affiliations:** 1 School of Health Sciences, National Open University of Nigeria, Lagos, Nigeria

Paper approved. Corrections noted and appreciated. 

 Please check line 8: Introduction...an n additional site (just wondering if the 'n' is a typographical error

I have read this submission. I believe that I have an appropriate level of expertise to confirm that it is of an acceptable scientific standard.

# Peer review: Referee response for version 1

**DOI:** 10.5256/f1000research.11568.r22022

**Published:** 2017-04-20

**Authors:** Chris Zielinski

**Affiliations:** 1 Partnerships in Health Information (Phi) Programme, Winchester Centre for Global Health, University of Winchester, Winchester, UK

Interesting and useful paper. My quibbles are

  

1. With the numbers. In the abstract is says “1174 students from 100 countries have registered”. In the introduction, it says “1256 people, from 80 countries, have enrolled.” From the data presented (and the repetition in the Results paragraph) it appears that the first of these is correct – although I have some doubts given the round numbers (100, 80) of countries cited.
2. In methods it merely states that “A suite of courses was developed” – it would be interesting to know by whom the suite of courses was developed and when. Some background would be welcome.
3. The second paragraph in the Results has some lapses in language (“a number of questions were asked to the students”, “the majority of students came as a recommendation from someone else”. The whole paragraph should be redrafted.
4. There is a typo in Box 1 in the longest quote – at the top of the column “but does not” should be “but does not”.
5. There is some repetition throughout – for example, the second paragraph of the discussions repeats some of the second para of the results.

I have read this submission. I believe that I have an appropriate level of expertise to confirm that it is of an acceptable scientific standard.

# Response

**Published:** 2017-04-26

**Authors:** Richard Heller

**Affiliations:** People's Open Access Education Initiative (Peoples-uni), Australia

*Response: The confusion has arisen due to the fact that the statement “1256 people, from 80 countries, have enrolled.”reflects enrolments in our courses for academic credit as part of the introduction to Peoples-uni, and are not results from the study we report in this paper.* *In order to try and clarify this issue, we have redrafted the section in the Introduction.*

*We have also added a new first paragraph to the Methods section, and redrafted paragraph 2 in the Results section.*

# Peer review: Referee response for version 1

**DOI:** 10.5256/f1000research.11568.r21231

**Published:** 2017-04-05

**Authors:** Michael Rowe

**Affiliations:** 1 University of the Western Cape, Department of Physiotherapy, Faculty of Community and Health Sciences, Bellville, South Africa

The institution seems like it is doing wonderful work and represents an interesting approach to open online courses. The courses offered by the institution seem useful and provide a learning opportunity for participants with an emphasis on those in developing countries.

 The structure of the courses could probably be improved by using pedagogical principles and learning theory with respect to design, but I think that is probably true of most open online courses. It also does not seem to fall within the scope of this research note, and I mention it only because so much attention is paid by the authors to the course design.

 This note represents what could possibly become a reasonable study of effectiveness of this innovative approach to open online courses, and I encourage the authors to build on this early work by designing a rigorous method and incorporating some analysis of the data.

I have read this submission. I believe that I have an appropriate level of expertise to confirm that it is of an acceptable scientific standard, however I have significant reservations, as outlined above.

# Response

**Published:** 2017-04-26

**Authors:** Richard Heller

**Affiliations:** People's Open Access Education Initiative (Peoples-uni), Australia

*Response: We have added a paragraph in the Discussion to discuss the pedagogy, and have added a sentence suggesting the need for a more rigorous evaluation to* *measure effectiveness.*

*.*

# Peer review: Referee response for version 1

**DOI:** 10.5256/f1000research.11568.r21124

**Published:** 2017-03-20

**Authors:** Jane-frances Obiageli Agbu

**Affiliations:** 1 School of Health Sciences, National Open University of Nigeria, Lagos, Nigeria

This study on "Open Online Courses in Public Health: experience from Peoples-uni" is very insightful as it shares findings of a unique Public health course offered by Peoples-uni. 

**Observations**

 Grammatical expression was a bit poor. I suggest paper should be reviewed by an English expert for better clarity.

 Flowery languages in the text should be discouraged (eg..."an army of volunteer tutors", "a sister site", "a suite of courses was developed" etc.

 Result: This statement is not clear "S *ome students enrolled in more than one course and we report on enrolments in **1*** ***597 courses*" **Is this referring to student population or number of courses enrolled in?

 Furthermore, please take note of typographical errors (eg, OOCs instead on MOOCs).

I have read this submission. I believe that I have an appropriate level of expertise to confirm that it is of an acceptable scientific standard, however I have significant reservations, as outlined above.

# Response

**Published:** 2017-04-26

**Authors:** Richard Heller

**Affiliations:** People's Open Access Education Initiative (Peoples-uni), Australia

We have made changes to clarify some of the wording and language.
